# The Role of the FODMAP Diet in IBS

**DOI:** 10.3390/nu16030370

**Published:** 2024-01-26

**Authors:** Luisa Bertin, Miriana Zanconato, Martina Crepaldi, Giovanni Marasco, Cesare Cremon, Giovanni Barbara, Brigida Barberio, Fabiana Zingone, Edoardo Vincenzo Savarino

**Affiliations:** 1 Department of Surgery, Oncology, Gastroenterology, University of Padua, 35121 Padua, Italy; luisa.bertin.1@studenti.unipd.it (L.B.); miriana.zanconato@studenti.unipd.it (M.Z.); martina.crepaldi.3@studenti.unipd.it (M.C.); brigida.barberio@unipd.it (B.B.); fabiana.zingone@unipd.it (F.Z.); 2 Gastroenterology Unit, Azienda Ospedale-Università Padova, 35128 Padua, Italy; 3 IRCCS Azienda Ospedaliero, Universitaria di Bologna, 40138 Bologna, Italy; giovanni.marasco4@unibo.it (G.M.); cesare.cremon@aosp.bo.it (C.C.); giovanni.barbara@unibo.it (G.B.); 4 Department of Medical and Surgical Sciences, University of Bologna, 40126 Bologna, Italy

**Keywords:** FODMAPs, nutritional status, fructose, lactose, fructans, galactans, glucose, irritable bowel syndrome

## Abstract

The low FODMAP (fermentable oligosaccharide, disaccharide, monosaccharide, and polyol) diet is a beneficial therapeutic approach for patients with irritable bowel syndrome (IBS). However, how the low FODMAP diet works is still not completely understood. These mechanisms encompass not only traditionally known factors such as luminal distension induced by gas and water but also recent evidence on the role of FOMAPs in the modulation of visceral hypersensitivity, increases in intestinal permeability, the induction of microbiota changes, and the production of short-chain fatty acids (SCFAs), as well as metabolomics and alterations in motility. Although most of the supporting evidence is of low quality, recent trials have confirmed its effectiveness, even though the majority of the evidence pertains only to the restriction phase and its effectiveness in relieving abdominal bloating and pain. This review examines potential pathophysiological mechanisms and provides an overview of the existing evidence on the effectiveness of the low FODMAP diet across various IBS subtypes. Key considerations for its use include the challenges and disadvantages associated with its practical implementation, including the need for professional guidance, variations in individual responses, concerns related to microbiota, nutritional deficiencies, the development of constipation, the necessity of excluding an eating disorder before commencing the diet, and the scarcity of long-term data. Despite its recognized efficacy in symptom management, acknowledging these limitations becomes imperative for a nuanced comprehension of the role of a low FODMAP diet in managing IBS. By investigating its potential mechanisms and evidence across IBS subtypes and addressing emerging modulations alongside limitations, this review aims to serve as a valuable resource for healthcare practitioners, researchers, and patients navigating the intricate landscape of IBS.

## 1. Introduction

Irritable bowel syndrome (IBS) is a persistent condition involving the intricate interplay between the gut and the brain and is one of the most common disorders of gut–brain interaction (DGBIs) [1]. Its prevalence varies from 3.8% to 12% in the general population, influenced by diagnostic criteria and geographical location, with a higher occurrence in females [2,3,4]. Diagnosis involves a targeted approach, incorporating a thorough medical history, examination, and a limited number of specific blood and stool tests to exclude the most frequent organic causes of gastrointestinal symptoms [5,6].

According to the Rome IV criteria, IBS patients exhibit chronic or recurrent abdominal pain (occurring at least one day per week in the last three months) associated with defecation and/or altered bowel habits. These features should occur in the absence of positive findings on recommended tests to rule out organic diseases (complete blood count, *C*-reactive protein, fecal calprotectin, and celiac serology) and symptoms of alarm. IBS is categorized into four subtypes: IBS with a predominance of constipation (IBS-C), IBS with a predominance of diarrhea (IBS-D), mixed bowel habits (IBS-M), or unclassified IBS-U [7].

Patients frequently describe additional symptoms and associated conditions, such as abdominal bloating or distension, overlapping upper gastrointestinal symptoms, and psychological issues [1,8]. Although not life-threatening, IBS significantly impacts patients’ quality of life, leading to increased healthcare referrals and reduced work productivity [9].

Although IBS has conventionally been perceived as either a motility disorder or a psychosomatic condition, our comprehension of its pathophysiology has undergone significant development in the past decade. Recent evidence suggests that infiltration of immune cells into the mucosal tissue, particularly mast cells and eosinophils, triggers the heightened release of pain-inducing mediators. This activation of sensory neurons contributes to visceral hypersensitivity and the manifestation of troublesome symptoms. The complex interplay between immune activation and a compromised gut barrier encompasses changes in microbiota as well as diet and psychological stress [10,11,12,13,14].

IBS is a multifactorial disorder characterized by numerous pathophysiological mechanisms, although its etiology remains unknown [11,15,16,17,18]. Despite the development of several targeted therapies in the last 20 years, most drugs demonstrate limited efficacy, and placebo response rates are high [19,20,21,22,23].

Given the limited success of pharmaceutical approaches, patients often explore alternative strategies. It is noteworthy to add that the success of alternative approaches is also linked to the fact that patients may prefer not to take medications, especially for extended periods. More than 80% of individuals with IBS symptoms note connections to food and frequently modify their diets as a means to enhance their condition, opting for dietary adjustments (e.g., gluten-free diets (GFDs) and elimination diets based on IgG antibody testing) instead of resorting to medications, although their efficacy lacks robust supporting data [24,25,26,27,28,29].

Among the available options, the low fermentable oligosaccharide, disaccharide, monosaccharide, and polyol (FODMAP) diet emerges as the most evidence-supported dietary intervention for IBS. FODMAPs can be found in different concentrations within specific fruits, vegetables, legumes, dairy products, artificial sweeteners, and nuts [30].

The implementation of a low FODMAP diet can be approached through two methods: top-down and bottom-up. Top-down approaches involve three phases, starting with FODMAP restriction, followed by the gradual reintroduction of previously eliminated foods to assess tolerance, and concluding with personalization to create a modified FODMAP-containing diet based on individual tolerance. Conversely, the bottom-up approach begins by initially reducing foods rich in specific FODMAPs for a specified duration and then further restricting other foods if necessary. It is crucial to emphasize that the preponderance of evidence supports the efficacy of the top-down approach [31,32].

This narrative review aims to provide an overview of the mechanisms of FODMAP diets in IBS, identify the optimal settings for their application, and discuss potential disadvantages. In doing so, we aim to offer clinicians a comprehensive guide for understanding and implementing FODMAP diets in different IBS subtypes, thereby contributing to improve patient outcomes and quality of life.

## 2. Low FODMAP Diet

Researchers at Monash University in Australia, headed by Dr. Sue Shepherd and Prof. Peter Gibson, introduced the term FODMAP in 2005. They conceptualized FODMAPs as a categorization for particular fermentable carbohydrates capable of eliciting gastrointestinal symptoms, particularly in individuals with IBS [33]. In the two decades leading up to this introduction, evidence had surfaced suggesting a connection between poorly absorbed short-chain carbohydrates such as lactose, fructose, and sorbitol and the manifestation of symptoms associated with IBS [34].

In 2006, the first research trial confirming the efficacy of a low fructose/fructan diet reducing symptoms was conducted. This study involved 62 individuals with IBS and fructose malabsorption, with 74% reporting symptom relief on the prescribed diet [35]. 

In 2008, the first double-blind, randomized, placebo-controlled rechallenge trial investigating fructans and fructose in individuals with IBS was published, providing further validation for the efficacy of the diet in managing IBS. The study involved IBS patients who underwent a gradual increase in the intake of glucose, fructose, fructans, or a combination of the latter, with at least a 10-day washout period between challenges. The findings indicated that a notably higher percentage of patients in the fructose and fructans group reported insufficiently controlled and more severe symptoms compared to those receiving glucose [36].

Mechanistic investigations in 2010 explored the effects of FODMAPs in an ileostomy model and measured breath hydrogen levels in IBS patients and healthy participants on low and high FODMAP diets. The results indicated an osmotic effect of FODMAPs and their fermentative properties, contributing to symptoms such as bloating, distension, stomach pain, and flatulence [37,38].

These mechanistic findings align with the known pathophysiological processes of IBS, including visceral hypersensitivity and changes in gut microbiota. 

The current low FODMAP diet includes six carbohydrates and was formulated to diminish the consumption of all FODMAP categories by identifying low FODMAP alternatives within each food group. Published tables detailing the composition of various foods are available [39]. Additional strategies for minimizing FODMAP intake, such as adding lactase to food or taking it orally to reduce lactose levels in applicable foods, and combining glucose with foods high in free fructose, are also recommended for individuals with IBS [40].

It is believed that FODMAPs exhibit three common functional properties: limited absorption in the small intestine, being small and osmotically active molecules, and rapid fermentation by bacteria.

The poor absorption of FODMAPs involves several mechanisms. Firstly, factors such as the reduced activity of brush border hydrolases in some individuals (e.g., lactase), the absence of hydrolases for fructans and galactans, and the molecular size being too large for simple diffusion (e.g., polyols) all contribute to the challenges in absorption [41]. Additionally, the slow and low-capacity transport mechanisms across the epithelium contribute to inefficient absorption. Indeed, fructose is primarily absorbed in the small intestine through two carriers belonging to the glucose transporter family (GLUT). Notably, GLUT5 is dedicated to fructose transport, while GLUT2 facilitates the transport of fructose, galactose, and glucose [42]. When consumed in conjunction with glucose, fructose exhibits enhanced absorption. Nevertheless, an imbalance favoring excess fructose over glucose hampers absorption, prompting fermentation by colonic gut microbiota [43,44,45]. The absorption dynamics are influenced by factors like dosage, intestinal transit speed, luminal glucose content, and individual absorptive capacity through the glucose transporters GLUT 2 and GLUT 5. 

The osmotic property is exemplified by synthetic FODMAP lactulose, also known as 1,4 beta galactoside-fructose. Lactulose, an artificial disaccharide made up of galactose and fructose that is not absorbed by the body, increases the water content of the small bowel which subsequently influences gut motility. Through this mechanism it triggers a laxative effect when given in adequate amounts [46]. 

The speed of bacterial fermentation is influenced by the length of carbohydrate chains, with oligosaccharides and sugars undergoing rapid fermentation compared to polysaccharides [47]. 

All FODMAPs are commonly grouped together because of their comparable physiological effects, but this categorization is not entirely accurate. While they can collectively induce an osmotic effect, it is important to note that, for instance, fructose and polyols produce a greater osmotic effect compared to galactooligosaccharides (GOS) and fructans. On the contrary, oligosaccharides demonstrate more noticeable fermentative effects because of their restricted absorption, in contrast to fructose and polyols that are absorbed through the small intestinal wall. Imaging provides evidence of heightened distension in the small intestine with fructose, mannitol, or sorbitol [40,41]. 

**Figure 1:**
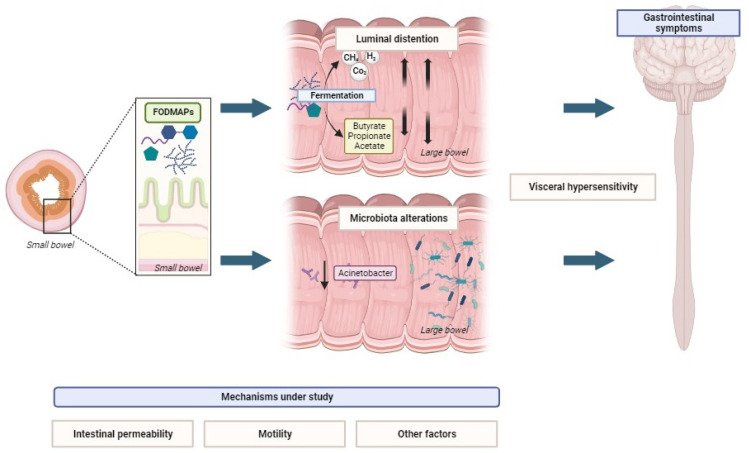
Hypothesized mechanisms of FODMAP-induced symptoms in individuals. Created with Biorender.com. Last accessed: 23 January 2023.

Various hypotheses have been suggested to explain the potential mechanisms by which FODMAPs induce symptoms in specific individuals. They are synthesized in Figure 1.

### 2.1. Luminal Distention by Gas and Water

Luminal distension triggered by FODMAPs has been linked to symptom development, with two contributing factors: an increase in small bowel water content and elevation in large bowel gas production.

The small intestinal hypothesis posits that unabsorbed and osmotically active carbohydrates attract water into the small intestine, as evidenced in lactose studies involving lactase deficiency [48]. This increased water content in the small bowel accelerates the transit to the cecum. As mentioned in this review, a single-blind crossover trial revealed that a high FODMAP diet increased bowel water content in 12 patients with ileostomy. However, this remained below the quantity tolerated by healthy individuals [38,49]. MRI data illustrated a substantial increase in small intestinal water content following the consumption of mannitol, fructose, or fructans compared to glucose, potentially resulting in distension and symptoms such as bloating [41,50]. Nevertheless, contradictory MRI data propose reduced small intestine water content in IBS-D patients, compared to their healthy counterparts [50,51]. 

FODMAPs that remain intact as they reach the distal portions of both the small and large intestine serve as a nutrient source for specific microbial species, undergoing fermentation generating gases (hydrogen, methane, and carbon dioxide) and causing intestinal distension. However, MRI imaging studies have shown comparable gas and bowel distension levels in both patients and healthy controls following fermentable carbohydrate intake [41]. The outcomes of a crossover study involving both healthy individuals and those with IBS revealed that the controls exhibited fewer symptoms after drinking a solution containing inulin, fructose, or glucose despite similar MRI parameters and breath hydrogen responses [1]. This finding challenges the direct association between gas production and symptom intensity in IBS patients. The underlining reason for symptoms in IBS patients is thought to be linked to hypersensitivity to distension, mirroring the symptom generation observed in lactose malabsorption [52]. In fact, a strong correlation exists between the hydrogen gas in breath and reports of symptoms like bloating and pain in lactose-intolerant IBS patients, although the level of hydrogen gas does not correlate precisely with symptom severity. The reduction in the consumption of foods rich in specific short-chain carbohydrates can impact hydrogen and methane production, as well as luminal distension [53]. Diets with varying FODMAP content lead to significant differences in breath hydrogen production, with elevated levels on the high FODMAP diet found for both healthy controls and IBS patients [37]. The low FODMAP diet correlated with reduced breath hydrogen, an effect reversed by oligofructose supplementation [54]. 

### 2.2. Visceral Hypersensitivity

Evidence of visceral hypersensitivity in a subset of IBS patients suggests that the same stimulus magnitude may result in varying degrees of symptom response depending on their sensory threshold [55,56,57]. 

In a study conducted by Evans et al., IBS patients underwent a hydrogen breath test after consuming 10 g lactulose and 25 g fructose plus sorbitol. Jejunal motility was assessed using 24-h manometry and visceral sensation using a balloon distention model. Although both jejunal dysmotility and hypersensitivity were altered, the study did not find a significant association between dysmotility, carbohydrate malabsorption, and symptom provocation [58]. 

Studies on rodents have indicated that a high FODMAP diet could potentially result in dysbiosis, dysfunction of the colonic barrier, recruitment and activation of mast cells, and the development of visceral hypersensitivity [59,60,61]. Singh et al. demonstrated, using mast cell-deficient rodents with or without mast cell reconstitution, that the loss of the colonic barrier induced by a high FODMAP diet relies on toll-like receptor 4 (TLR4)-dependent mast cell activation [60]. In a previous 2018 study, the same authors fed rats a high FODMAP diet, resulting in increased fecal Gram-negative bacteria, elevated lipopolysaccharides (LPS), and induced intestinal pathology, evidenced by barrier dysfunction and visceral hypersensitivity. The barrier loss was reversed by a low FODMAP diet [61]. The proposed mechanism involves an abundance of Gram-negative bacteria induced by a high FODMAP diet, leading to increased luminal LPS. LPS activates mast cells via TLR4, releasing molecules like tryptase, histamine, and prostaglandin E2, thereby increasing intestinal permeability and visceral sensitivity [60]. Kamphuis et al. also studied the effect of FODMAPs in rodents, and found that visceral hypersensitivity is linked to elevated expression of the advanced glycosylation end product-specific receptor and is alleviated in the presence of an antiglycation agent [59]. Further research is needed to understand the mechanism through which specific food components affect the production of neuroactive mediators by the gut microbiota.

### 2.3. Increased Intestinal Permeability

Enhanced intestinal permeability is suggested as a potential factor, with ongoing research exploring this aspect [33,61,62]. In a controlled, single-blind study where patients with IBS were randomized to either a low or high FODMAP diet for 3 weeks, it was found that individuals on the low FODMAP diet exhibited reduced urinary histamine levels [63]. This implies a potential restoration of mucosal integrity. Urinary histamine is considered a surrogate marker for mast cell activation in patients with DGBI, given its association with increased mast cell activation and its role in sensitizing nociceptive and enteric neurons [10,13,63]. 

Prospero et al. performed a trial in IBS-D patients who underwent 12 weeks of a low FODMAP diet to evaluate their clinical, nutritional, biochemical, and psychological status before and after. The authors found that markers of intestinal mucosal integrity, including serum levels of intestinal fatty acid-binding protein, diamine oxidase, and serum zonulin (though not yet validated [64]), significantly decreased after the low FODMAP diet [14,65]. Singh et al., utilizing a mouse model in vitro, demonstrated that fecal supernatant from individuals with IBS on a high FODMAP diet stimulates mast cells more significantly than that from healthy controls. This effect is mitigated in the absence of TLR4 and after adopting a low FODMAP diet [60].

### 2.4. Microbiota Alterations, SCFA Production, and Metabolome

Although different studies have associated IBS with dysbiosis, the evidence supporting its pathophysiological role in IBS remains controversial. Certain studies observed variations in the gut bacterial signature, while others were unable to reproduce these results, indicating that a distinct bacterial microbiota might be evident only in a subset of IBS patients [66,67,68,69,70]. A systematic review of case–control studies, encompassing 24 studies that compared gut microbiota in patients with IBS and healthy controls, recognized diversity in microbiota composition between these two groups. However, the review also underscored inconsistent results among studies, a lack of standardized descriptions of interventions, and limited sample sizes, emphasizing the need for further research in this complex field [71]. 

To analyze the impact of the low and high FODMAP diets on human microbiota, a systematic review was published in 2020 [72]. The review encompassed seven randomized controlled trials (RCTs) involving patients with IBS, revealing that FODMAP restriction resulted in a relatively consistent overall community diversity but a lower abundance of *Bifidobacteria* and its phylum *Actinobacteria*. The metabolic capabilities of *Bifidobacteria*, allowing them to degrade various fibers, including fructans, may account for these effects [73]. In fact, selective stimulation of Bifidobacteria growth has been observed with fructan supplementation, at least in healthy individuals [74]. However, the studies included in the meta-analysis had some downsides: they employed diverse methodologies, had small sample sizes, and only investigated short-term FODMAP restriction. 

Intraluminal intestinal fermentation by colonic bacteria results in the generation of gases (hydrogen, methane, and carbon dioxide), as well as SCFAs and branched-chain fatty acids (BCFAs) as secondary by-products. Protein fermentation can give rise to the formation of BCFAs [75,76]. SCFAs, including propionate, butyrate, and acetate, are products of the bacterial metabolism of dietary fiber, showcasing numerous advantageous effects. Specifically, butyrate serves as the primary energy source for the colonic epithelium and plays a crucial role in maintaining its health [77]. Moreover, propionate and acetate may have systemic immunomodulatory and epigenetic impacts. An abundance of SCFAs has been considered to potentially present a risk to epithelium homeostasis [78]. Furthermore, the effects of SCFAs on visceral sensitivity are subject to debate, with the potential to stimulate the liberation of 5-hydroxytryptamine from the intestinal mucosa [79]. This stimulation may promote the initiation of high-amplitude propagated colonic contractions, thereby accelerating intestinal transit [80]. 

While the presumed reduction in fermentation in the low FODMAP diet should theoretically lead to a decrease in SCFA production, inconsistent effects of FODMAPs on fecal SCFAs have been observed. Additionally, their impact on plasma concentrations has not been investigated before [81,82,83,84,85]. The recent systematic review by So et al. found no differences in total fecal SCFA concentration between the low FODMAP diet and control diets, nor differences in the fecal concentrations of specific SCFAs or fecal pH [83]. 

Animal studies and clinical trials involving humans suggest that implementing a low FODMAP diet may lead to elevated generation of branched-chain fatty acids (BCFAs), likely attributable to heightened protein fermentation. However, research on patients with IBS-D following a low FODMAP diet is inadequate, and there is a lack of concurrent measurements of inflammatory biomarkers and fecal BCFAs. BCFAs are capable of oxidation in the absence of butyrate and function as an alternative energy source [86].

The role of BCFAs and SCFAs in IBS pathogenesis or low FODMAP diet symptom management remains unclear without new evidence.

Growing interest in food metabolomics aims to understand the complex diet–patient interaction, recognizing a significant metabolome connection to the microbiome [87,88,89,90]. However, attributing metabolite origin to host versus microbiota is challenging due to extensive co-metabolism. There is a scarcity of studies investigating the collective influence of gut microbiota and metabolites in individuals with IBS and FODMAPs. A study performed in 2017 by McIntosh et al. showed that patients in the low FODMAP group had eightfold-reduced urinary histamine levels, with a strong correlation between *Porphyromonadaceae* spp. and urinary histamine [63]. Nordin et al. recently investigated the influence of FODMAPs on gut microbiota and their interaction with the metabolome and clinical response in individuals with IBS. During a diet excluding both gluten and with minimal FODMAP consumption, IBS patients underwent single challenges of gluten, FODMAPs, and a placebo, followed by individual challenges of each component. Significant changes in the composition of the gut microbiota were observed in the FODMAP group but not in the gluten group, correlating with alterations in plasma metabolites. Specifically, the genera *Agathobacter*, *Anaerostipes*, *Fucicatenibacter*, and Bifidobacterium exhibited associations with increased levels of phenolic-derived metabolites and 3-indolepropionate. However, there was only a weak correlation between microbiota changes and IBS symptoms [91].

### 2.5. Motility

Few studies have investigated FODMAPs’ impact on gastrointestinal motility. 

Animal studies have demonstrated that hyperosmolar solutions of mannitol can stimulate duodenal motility, independent of changes in gastric inhibitory peptide or insulin [92,93]. A limited study on polyol consumption and gastrointestinal motility by Evans et al. found no association between jejunal dysmotility, carbohydrate malabsorption, and symptom provocation in female patients with IBS [58].

To study this subject, Masuy et al. conducted an RCT, administering fructans or glucose to both IBS patients and healthy controls. Continuous manometric measurements over three hours revealed varying upper gastrointestinal motility responses between carbohydrates. The IBS patients exhibited higher sensitivity to fructan infusion, as evidenced by elevated gastrointestinal symptom scores [94]. 

This result was affirmed in a recent double-blind crossover investigation conducted by Wu et al. In this study, the authors conducted brain and abdominal MRIs, along with symptom evaluations, on both IBS patients and healthy controls after intragastric infusion of a solution containing fructans, glucose, and saline. Despite fructans inducing similar increases in small bowel motility and colon gas and volume in both groups, the heightened symptom responses in IBS were linked to altered brain responses in regions associated with pain [95].

## 3. Symptom Improvement with Low FODMAP Diets

In the last few years, physicians have suggested to some IBS patients simple changes in dietary behavior, aiming to offer beneficial effects on symptoms. Indeed, the low FODMAP diet has gained increasing popularity based on numerous positive interventional trials.

### 3.1. Low FODMAP Diet Implementation

Different studies have already reported that symptom recurrence is more related to oligofructose or fructose than glucose [36]; in addition, individual FODMAPs (lactose, fructose, fructooligosaccharides, and sorbitol) induce abdominal symptoms that are commonly described by IBS patients: bloating, pain, nausea, and an increase or decrease in stool frequency, as we have already explained in the previous paragraphs [45].

In a small study, breath testing made it possible to find that the most common cause of functional bloating and gas-related symptoms was fructose intolerance. Hence, it was demonstrated that about 65% of patients presented carbohydrate malabsorption, and improvement of symptoms was found in more than 80% of patients after a month of dietary restriction. Complete improvement was registered in 50% of patients after 1 year of treatment [96].

Physicians have long been aware of lactose and polyols as a trigger for gastrointestinal symptoms, and dietary restriction can help to manage symptoms that are related to lactose malabsorption [47].

Multiple clinical trials have demonstrated the efficacy of a low FODMAP diet in IBS management, particularly abdominal bloating, with an approximate improvement in 50–75% of IBS patients [97].

Several international guidelines acknowledge the potential of dietary interventions, specifically endorsing a low FODMAP diet as a treatment option for IBS [5,6,98,99,100]. In most cases (i.e., UEG, Italian, BSG, and Asian guidelines), a low FODMAP diet is suggested as a second-line treatment for IBS [6,100,101,102]. The quality of evidence in these guidelines is classified as weak, however, because of the lack of sound RCTs. 

In addition, most guidelines suggest that the course of the diet and the food reintroduction according to tolerance should be supervised by a trained dietitian [101,103]. In some studies, including real-life studies, above all during the SARS-CoV-2 pandemic, apps and remote instructions were tried in order to lead patients to follow a correct diet and resulted in being useful to help patients improve their symptoms with a distant supervisor [104]. As for the guidelines’ recommendations, the Italian guidelines for the management of IBS suggest a traditional diet as the first line approach, followed by a low FODMAP diet in case of failure [6], while UEG and the ESNM recommend the use of a low FODMAP diet in patients with IBS-D, especially when other kinds of treatment have failed [5]. The Asian guidelines specifically highlight the lack of studies regarding the role of a low FODMAP diet in the management of IBS in the Asian population [99].

**Figure 2:**
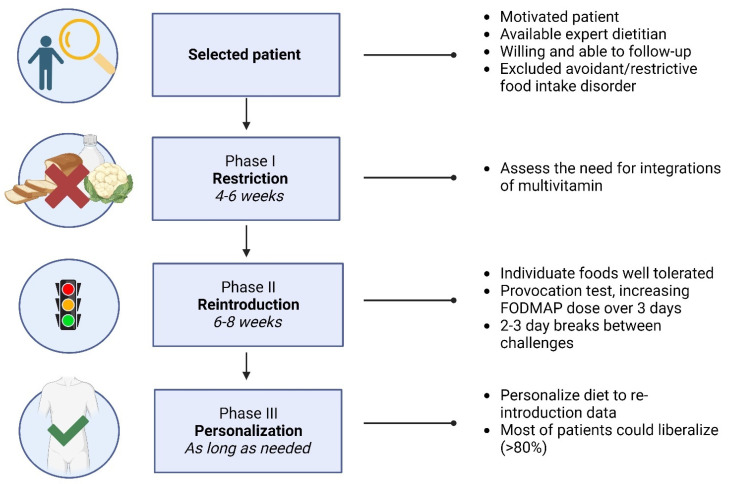
Process of low FODMAP diet implementation. Created with Biorender.com. Last accessed: 31 December 2023.

Furthermore, AGA best practice advice not only suggests the use of a low FODMAP diet as a therapy for IBS but also describes the “top-down” approach, divided into the three phases previously explained in this review (restriction, the reintroduction of FODMAP foods, and then personalization) [102]. The process of low FODMAP implementation, as described by different guidelines, can be seen in Figure 2. 

The low FODMAP diet can be useful for patients but also elaborated, and it is potentially more expensive than other dietary advice. Furthermore, it is not well known if patients with meal-related symptoms are assuming an inadequate diet, with less nutritional intake, so the dietitian indication could help to obtain a balanced diet in the personalization phase. Because of these reasons, GI physicians should be helped by a dietitian in a multidisciplinary team. GI dietitians can be helpful in screening patients with IBS for disordered eating patterns, food allergies, and food intolerances.

It is important that the patient reports symptom improvement during the restriction phase; otherwise, the low FODMAP diet should be discontinued.

### 3.2. Efficacy Compared to Other Diets and Other Treatments for IBS

**Figure 3:**
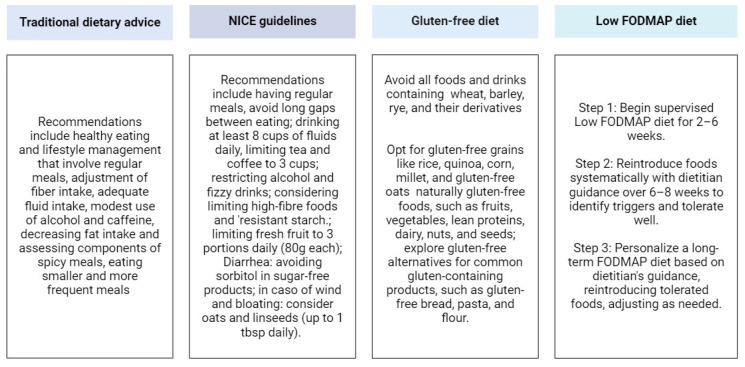
Main recommendations of dietary treatments for IBS. Created with Biorender.com. Last accessed: 31 December 2023.

The low FODMAP diet was compared to other dietary approaches proposed in the past, including TDA (traditional dietary advice), habitual diet (i.e., the Mediterranean diet and the Australian diet), BDA (British Dietetic Association) and NICE (National Institute for Health and Care Excellence) dietary advice, and the GFD. The main recommendations of these dietary treatments are represented in Figure 3.

A recent network meta-analysis evaluated the outcome of different diets including the low FODMAP diet, BDA/NICE dietary advice, and an alternative dietary regimen for global and single IBS symptoms. The results showed that the low FODMAP diet ranked first for global IBS manifestations. In addition, the NICE and BDA dietary suggestions had less promising results than the low FODMAP diet for bloating or distension severity. The low FODMAP diet determined a greater improvement for abdominal pain severity than the sham dietary regimen. Conversely, significant effects on bowel habits were not registered for the low FODMAP diet when compared to the other diets [32].

An RCT tried to suggest treatment with a low FODMAP diet or traditional dietary advice from NICE guidelines in a sample of 100 patients affected by IBS-D. The evaluated scores were IBS-Symptom Severity Score (IBS-SSS) and IBS-related Quality of Life (QOL) and, through them, an improvement in the patients’ condition was registered with both forms of dietary management. The low FODMAP diet brought greater benefits for the evaluated outcome: a more than 50 point reduction in IBS-SSS was achieved through the low FODMAP diet in 62.7% of patients vs. traditional dietary advice in 40.8% of patients [105,106]. 

The RCT by Halmos et al. evidenced the effects of a low FODMAP diet against the Australian diet on IBS patients and concluded that the first one was more effective in reducing overall gastrointestinal symptoms, especially bloating and pain [107].

In another small crossover RCT, Paduano et al. enrolled 42 patients with all types of irritable bowel syndrome and randomized them to low FODMAP diet, GFD, and Mediterranean diet groups, as an example of a “balanced diet”. Each dietary regimen demonstrated a reduction in GI symptoms (evaluated with a visual analogue scale, VSA) and an improvement in quality of life. However, the results have to be confirmed through a larger sample of patients. The authors underlined the low FODMAP diet’s ability to ameliorate bowel transit, highlighted by an improvement in the Bristol Stool Scale: this diet was the only one that could achieve the fourth grade of the scale in IBS-D patients [108].

A recent randomized trial tried to compare TDA, the GFD, and the low FODMAP diet in IBS-D and IBS-M patients regarding effectiveness and economic benefits and found that each diet had similar clinical efficacy, assessed by IBS-SSS: 42–58% of patients experienced a ≥50 point reduction with each piece of dietary advice. The patients had to complete questionnaires about the acceptability of dietary restriction questionnaires and QOL. The patients studied emphasized that TDA was easier to follow and incorporate into daily life than the low FODMAP diet and the GFD because it was less expensive (*p* < 0.01), made it less time-consuming to shop (*p* < 0.01), and had less impact when eating out (*p* = 0.03). In light of these findings, the authors suggest using the TDA in clinical practice and reserving the GFD and low FODMAP diet for selected cases [109]. These results were thereafter commented on by Staudacher et al. who complained about the fact that the response rate for the low FODMAP diet was set to 75% in the trial; conversely, the other RCTs reported the response rate of 50–52%. Moreover, they commented that TDA is not rigid and is designed to be personalized, so differences in TDA results through trials could be caused by the diverse evaluated populations and in the heterogeneous dietary regimen followed within the TDA definition. Finally, TDA (or indeed the GFD) does not give the possibility to allow the patients to manage their symptoms and does not have step-down phases [110]. Nevertheless, more studies need to confirm the previous sentences.

Only a few studies have compared the efficacy of a low FODMAP diet with other non-dietary treatments. 

An unblinded RCT demonstrated that 6-week treatment with a low FODMAP diet and Lactobacillus rhamnosus GG can be more efficacious than a Danish/Western diet in IBS patients [111].

Menees et al. compared the effects of the low FODMAP diet and psyllium treatment. The authors found that 4-week low FODMAP diet treatment was related to a significant improvement in mean FISI scores for stool consistency versus baseline (39.2 vs. 32.6, *p* = 0.02), whereas psyllium did not show a similar result (35.2 vs. 32.5, *p* = 0.22) [112].

A recent 8-week trial conducted in Belgium in the setting of primary care enrolled 459 IBS patients and randomized them to two groups of treatment: a low FODMAP diet group or an anti-spasmodic drug, otilonium bromide, group. The instructions were released via smartphone or tablet app, focused on reducing FODMAPs rather than strict elimination, and the study involved both IBS-D and IBS-C patients. After 8 weeks, the diet group showed a more frequent occurrence of the primary outcome (≥50 point improvement on a 500 point standardized IBS symptom scale) compared to the medication group (71% vs. 61%), with consistent results observed across the stool pattern subtypes [113].

Gut-directed hypnotherapy and yoga could be as effective as the low FODMAP diet in improving IBS symptoms after 6 months of treatment; this was demonstrated by two comparative trials [114,115].

### 3.3. Treatment Personalization

This kind of treatment can be personalized to the patient, who can tolerate and reintroduce foods that do not cause the onset of symptoms. This empowers individuals to take control of their symptoms and make informed decisions about their diet. Patients can follow a properly “modified low FODMAP diet”, and studies report the persistent benefit from it after 6–18 months [31,116].

Certain studies propose an alternative to the traditional low FODMAP diet, referred to as “FODMAP-gentle”, which follows a bottom-up approach. This method involves reducing the intake of specific foods with high FODMAP concentrations or targeting certain FODMAPs. In cases of non-response or partial response, stricter restrictions may be implemented. This approach might be suitable for specific populations, including individuals at risk of malnutrition, children, those with comorbidities negatively impacted by dietary changes (e.g., inflammatory bowel disease patients and pregnant women), or those with a reluctance or limited ability to adhere to or understand the dietary requirements [117]. 

This diet can be adapted to any different dietary preferences, such as vegetarian, vegan, and gluten-free options. This flexibility makes it accessible to a wider range of individuals.

However, as in the top-down approach, it is essential to avoid unnecessary restrictions, reintroducing high-FODMAP foods gradually as tolerated and maintaining a balanced diet in the long term.

In addition, many reintroduction trials have recognized only fructans, mannitol, and galactooligosaccharides as the most common in inducing the recurrence of symptoms, so it could be possible to think about a simplified version of a low FODMAP diet, but more studies confirming this evidence are needed [102].

In a recent RCT, individuals with IBS who responded positively to the low FODMAP diet were reintroduced to different groups: 100% fructose, 56% fructose/44% glucose, or 100% glucose and were administered four groups of doses (2.5, 5, 10, and 15 g) each day for 3 days. The majority of responders demonstrated tolerance to the 15 g sugar dose, leading the authors to conclude that higher doses are essential for a comprehensive assessment of tolerance [118]. 

Moreover, physicians need further evidence from RCTs, regarding the II and III phases (the reintroduction and personalization phases, respectively) of a low FODMAP diet, together with data on adherence and long-term effectiveness.

## 4. FODMAP Diet Response Markers

Most studies have concentrated on assessing the effectiveness of the low FODMAP diet in the IBS population, showing success rates ranging from 50 to 75% as described in the previous paragraph. Initiatives have been undertaken to improve efficacy rates [119] and identify potential predictors of response, including identifying subsets of IBS patients in whom the diet is more likely to be effective [32]. In a recent cross-over trial, the authors found that clinical responders exhibited inclinations toward a more severe IBS profile (using IBS-SSS) and higher levels of anxiety at baseline [120]. However, more studies are needed to confirm this finding.

### 4.1. Breath Test

In a recent limited pilot investigation, the positive FODMAP meal challenge test (indicated by breath H2 levels exceeding 10 PPM above baseline accompanied by symptoms after consuming a high FODMAP meal) demonstrated sensitivity, specificity, and diagnostic accuracy of 78.6%, 66.6%, and 75.6%, respectively, in forecasting the response to a low FODMAP diet [121].

### 4.2. Microbiota Analyses

The potential predictors of treatment responses in the context of commencing a low FODMAP diet include the composition and richness of specific bacteria [122,123,124,125].

In 2015, Chumpitazi et al. conducted a double-blind, crossover trial involving pediatric patients with IBS, in which the participants were randomly assigned to either a low FODMAP diet or typical American childhood diet. Those responding positively to the low FODMAP diet exhibited an increase at baseline in taxa known for greater saccharolytic metabolic capacity within the family *Bacteroidaceae* (e.g., *Bacteroides*), order *Clostridiales* (e.g., *Ruminococcaceae*, *Dorea*, and *Faecalibacterium prausnitzii*) and the family *Erysipilotrichacea*.

Numerous studies have highlighted the significance of elevated colonic methane and SCFA production, coupled with increased saccharolytic fermentation activity [126]. These findings underscore the crucial role of the gut microenvironment in shaping responses to dietary interventions. Vervier et al. identified two distinct subtypes of microbiota in individuals with IBS, with the “pathogenic-like” subtype (enriched in Firmicutes and containing fewer Bacteroidetes species) exhibiting a more substantial response to the low FODMAP diet [123]. The microbiome of IBS individuals following a FODMAP diet was also analyzed with the utilization of a commercial “dysbiosis test” to analyze bacterial DNA profiles. Valeur et al. observed higher levels of Actinobacter and Streptococcus at baseline in responders, whereas Bennet et al. indicated lower levels of these microorganisms in responders at baseline [122,127]. These conflicting outcomes concerning individual microbial species in predicting responses to the low FODMAP diet need to be clarified in future studies.

The discrepancies in the findings can be attributed to substantial heterogeneity in methodology, encompassing not only the method employed to analyze the microbiome but also other factors such as the study design, the included population (e.g., different Rome Criteria-classified IBS), and defining the efficacy of a low FODMAP diet.

### 4.3. Fecal and Urinary Metabolites

Volatile organic compounds play roles as intermediaries or endpoints in metabolic pathways, offering insights into various aspects of colonic metabolism. Although differences in fecal volatile organic compound profiles between individuals responding and those not responding to the low FODMAP diet have been demonstrated, the identification of specific metabolites predicting responses is still pending [128]. Specific profiles of fecal volatile organic compounds have exhibited notable accuracy in forecasting responsiveness to the low FODMAP diet [128]. While a Swedish study effectively anticipated responsiveness to the low FODMAP diet through fecal bacterial profiles, a recent UK study did not, highlighting the uncertainty in this domain and underscoring the necessity for further research on the effect of the low FODMAP diet on the microbiome of the gut.

A recent blinded RCT led by Wilson et al. involved patients with IBS undergoing a sham diet or a low FODMAP diet and revealed that fecal metabolites at baseline (higher faecal propionate and cyclohexanecarboxylic acid esters) and urine metabolites were able to differentiate patients responding to the low FODMAP diet, aligning with previous findings [126,128]. In contrast to earlier studies suggesting that fecal microbiota could predict a response to the low FODMAP diet, this trial did not observe such a correlation, although it is worth noting that the sample sizes for comparison were small [129].

## 5. Downsides of the Low FODMAP Diet

While the low FODMAP diet has shown notable success in alleviating IBS symptoms for a substantial proportion of IBS patients, it is essential to critically examine its potential disadvantages and limitations. This section will explore various aspects that underscore the challenges and drawbacks associated with the application of the low FODMAP diet. From the necessity for professional guidance by dietitians to the unpredictable nature of individual responses, concerns related to microbiota, nutritional deficiencies, and issues in precisely quantifying FODMAP content in food products will be scrutinized. Furthermore, the potential impact on constipation, the association with eating disorders, and the scarcity of long-term data will be addressed. 

Despite its efficacy in symptom management, acknowledging these limitations is crucial for a comprehensive understanding of the broader implications and potential pitfalls of the low FODMAP diet.

### 5.1. Social and Lifestyle Challenges

In particular, the first phase of the low FODMAP diet may pose challenges in social situations and require meticulous meal planning, introducing lifestyle complexities for some individuals. In a secondary analysis of two RCT including 131 IBS patients, where the participants were randomly assigned to either a low FODMAP diet or a control diet, the authors found that a 4-week low FODMAP diet, guided by a specialist dietitian, did not significantly impact nutrient intake or diet diversity. However, it was observed to decrease diet quality compared to control diets. This parameter was calculated using the Healthy Diet Indicator and Healthy Diet Score [130]. Long-term observations indicated that the low FODMAP diet is not only more expensive than a usual diet but also affects aspects of quality of life, particularly in social eating situations [131,132]. 

In a recent head-to-head RCT comparing the efficacy and satisfactoriness of TDA, the low FODMAP diet, and the GFD in IBS patients, individuals reported that TDA was more cost-effective, required less time for shopping, and was easier to follow and implement in daily life compared to the low FODMAP diet [109]. 

### 5.2. Nutritional Guidance

Collaboration with a dietitian is essential to optimize outcomes, address potential complexities and increased food costs, explore alternatives to high FODMAPs, and enhance compliance. Nonetheless, in certain clinical environments where a dedicated dietitian is not readily accessible, it is not clear whether other educational methods could be employed. To answer this question, a recent RCT conducted by Dimidi et al. employed a three-arm design to compare education delivery methods—namely, booklets, apps, or dietitian assistance—for patients with DGBI following the low FODMAP diet. The authors found that individuals guided by a dietitian were more inclined to feel proficient in self-managing symptoms without requiring additional support [133]. 

Compliance with the low FODMAP diet appears to decline as individuals progress through its phases, as indicated by a case series of 81 patients published by Tuck et al. in 2019, involving patients with DGBIs. The decrease in adherence was observed gradually throughout the low FODMAP diet phases, with rates of 78% in phase 1, 48% in phase 2, and 40% in phase 3. Notably, adherence significantly improved when the low FODMAP diet was administered by a dietitian compared to other methods, with rates of 96% vs. 71% in phase 1 (*p* = 0.02), 70% vs. 39% in phase 2 (*p* = 0.02), and 65% vs. 29% in phase 3 (*p* < 0.01) [134]. 

However, additional research is needed to validate these findings.

### 5.3. Nutritional Deficiencies

Recent research has examined the nutritional adequacy of the low FODMAP diet. Studies on dietary intake have revealed inconsistencies, with the most common deficiencies occurring in fiber due to reduced carbohydrate intake. Excessive exclusion of dairy products may lead to decreased calcium intake, while deficiencies in vitamins such as B1, B2, B9, and D are associated with a relevant restriction of vegetables and fruits in the diet [135,136,137]. Iron deficiency has also been documented [138]. Lower energy consumption with the low FODMAP diet could result in weight loss [136]. Although long-term data on the effects of the low FODMAP diet are limited, proper monitoring by health professionals can help mitigate the risks of nutritional deficiencies [116,139].

An RCT published in 2015 compared the energy intake of the low FODMAP diet and TDA and found a reduction in energy with both diets after 4 weeks, greater with the former [140].

A post hoc analysis of an RCT comparing the modified NICE diet with the low FODMAP diet at 4 weeks revealed a decrease in daily kilocalorie consumption, along with reduced baseline micronutrients with the low FODMAP diet, although only changes in riboflavin were significant when adjusting for calorie intake [138]. An RCT published by Staudacher et al. in 2017 found no difference in the total number of calories and macronutrient intake between the low FODMAP diet and a sham diet [141].

The evidence on the low FODMAP diet in the long-term, however, suggests that the low FODMAP diet may be nutritionally adequate. Recent studies have demonstrated no significant difference in nutritional adequacy between the low FODMAP diet and a habitual diet when long-term data where analyzed [132,142]. Although total energy and macronutrient intake were reduced following the low FODMAP diet at long-term follow-up, this may be attributed to altered dietary habits in individuals with IBS, as many fail to meet their recommended dietary values [130,138]. The primary cause of deficiencies in the IBS population may be attributed to inadequate dietary counseling and self-imposed restrictions.

### 5.4. Microbiota Alterations

The connection between food and the microbiota is intricate and mutually influential, with each impacting the other [143]. While the reduction in carbohydrates, as observed in the positive symptomatic effect of the low FODMAP diet, may be beneficial for individuals with IBS, it could potentially have adverse effects on the gut commensal microbiome. This negative impact results from decreased fiber intake and the limited availability of fructans associated with the diet, which typically supports the establishment of beneficial microbial strains within the intestinal niche [144].

*Bifidobacterium*, a crucial producer of SCFAs like butyrate as previously described in this review, shows lower abundance in humans with more severe IBS symptoms [71,145,146,147]. The decline in Bifidobacteria observed serves as a deterrent to endorsing the prolonged adherence to a strict low FODMAP diet. Evidence indicates that, following the reintroduction and personalization of FODMAPs, the abundance of Bifidobacteria may revert to baseline levels [148]. Despite attempts to prevent short-term alterations, such as concurrent supplementation with a Bifidobacteria-containing probiotic, challenges persisted, and fiber supplementation did not yield the same effect [141,149].

### 5.5. Defining the Low and High FODMAP Content of Food

Understanding the FODMAP content of foods has been challenging and is an evolving process, initially characterized by patchy and limited knowledge [39,150,151]. 

For distinguishing high from low FODMAP foods, a suggested daily therapeutic target of 12 g of total daily FODMAP intake has been proposed. A recent systematic review and meta-analysis established that the collective consumption of FODMAPs within the population, regardless of health status, is estimated to be around 19.86 grams per day [152]. In 2021, Rej et al. conducted a literature review, encompassing nine studies, and found that the majority of participants achieved a total FODMAP intake of less than 12 g/day during the strict phase of the low FODMAP diet. Furthermore, the intake of fructans appeared to decrease to less than 2.2 g/day after the strict phase, and this reduction was sustained in the long term [153]. In 2017, McMeans et al. found discrepancies among three commonly used U.S.-based low FODMAP food lists, with an overlap of less than 50% in listed foods [154]. Martin et al. more recently reported that 22.6% of the 204 foods listed varied in classification across the 10 studies included [155]. Although the low FODMAP diet has demonstrated efficacy in individuals with IBS, particularly in the short term, the optimal threshold for achieving a total reduction in FODMAPs is yet to be determined.

**Figure 4:**
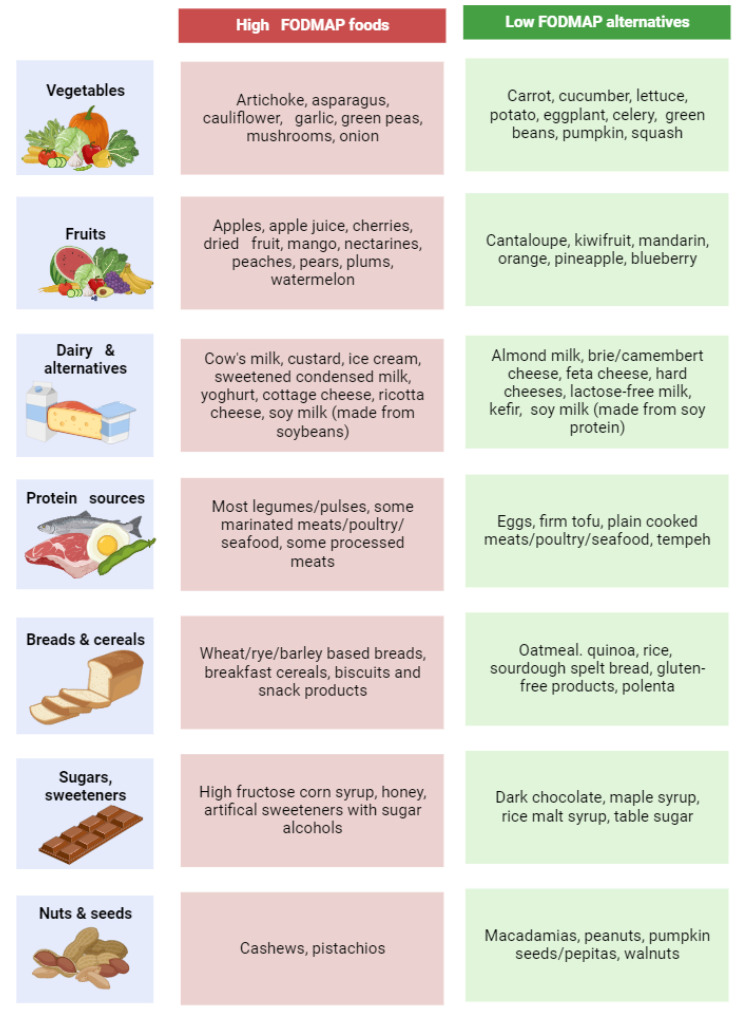
High FODMAP foods and low FODMAP alternatives. Created with Biorender.com. Last accessed: 23 January 2024.

Over the past decade, the Monash University Department of Gastroenterology has conducted extensive research to quantify the FODMAP composition of various foods, covering diverse categories such as fruits, vegetables, grains, cereals, dairy, meats, beverages, and condiments [39,150,151]. This ongoing effort has resulted in a comprehensive database, accessible through the Monash University low FODMAP diet app, which is regularly updated and available in multiple languages, facilitating global access to accurate FODMAP data for diet implementation. Suggested low FODMAP alternatives are represented in Figure 4.

### 5.6. Constipation

Another possible drawback of the low FODMAP diet is the restricted intake of fibers, which may worsen constipation [156]. Bellini et al. observed that fiber deficiency is relatively common among individuals following a low FODMAP diet, while Sultan et al.’s review emphasized conflicting results in previous studies [116,136]. In a recent randomized crossover trial led by So et al., individuals with IBS experienced three variations of the low FODMAP diet, distinguished solely by their fiber content. The study revealed that the addition of fiber during the low FODMAP diet did not alter patients’ perception of bowel movements. However, it did lead to the normalization of water stool content and colonic transit compared to the control diets [157].

### 5.7. Eating Disorders and Psychiatric Comorbidities

The constraints imposed during the initial phase of the low FODMAP diet may have adverse effects on the emotional well-being of patients. Anxiety, stemming from concerns about potential exacerbation of IBS symptoms and subsequent dietary restrictions, appears to be associated with the development of eating disorders [116]. Specifically, avoidant–restrictive food intake disorder and orthorexia nervosa are the most prevalent eating disorders linked to the low FODMAP diet [116,136]. Therefore, it is recommended to avoid restrictive diets, such as the low FODMAP diet, in individuals with eating disorders [158]. Pre-screening for malnutrition is also advised.

Moreover, cognitive limitations and significant psychiatric conditions can impede a patient’s ability to identify consistent food triggers, adhere to a restrictive diet, or accurately report clinical responses.

### 5.8. Limited Long-Term Data

While short-term studies offer evidence of the effectiveness of low FODMAP diets in symptom reduction, long-term data remain scarce, giving rise to concerns regarding potential nutritional deficiencies and the prolonged impact on the gut microbiome.

Adherence to the low FODMAP diet tends to decrease as individuals progress through its phases, as indicated by the previously mentioned case series of 81 patients with functional gastrointestinal disorders, but additional evidence is necessary to validate this observation [134].

To date, there is limited evidence from long-term studies supporting the efficacy of a low FODMAP diet. Real-world research indicates that adherence issues during the restriction phase were observed in less than 10% of patients [159]. During an extended observational study spanning an average follow-up period of 15.7 months, notable improvements in symptoms, such as the alleviation of abdominal pain, bloating, flatulence, and diarrhea, were observed. The reported adherence rate during this period was 76% [160]. Similarly, an RCT involving 74 IBS patients demonstrated sustained symptom response at 6 months for 82% of those on a low FODMAP diet [114].

Recent research conducted in the UK has suggested persistent symptom relief through the low FODMAP diet during extended follow-up periods, with therapeutic effects reported in as many as 60% of patients, averaging a follow-up duration of 44 months [132,142]. Additionally, between 65% to 82% of individuals following a low FODMAP diet transition to the personalization phase during long-term follow-up [132,134,142].

Further studies evaluating the low FODMAP diet over the long term are still required. 

## 6. Conclusions

The low FODMAP diet has become a valuable strategy in alleviating the symptoms of IBS and offering relief to those affected. Contemporary best practices in IBS management endorse a model of care which incorporates medical treatment, dietary modifications, and psychological therapy. This comprehensive approach, delivered by a multidisciplinary team, aims to empower patients for effective self-management over time.

The low FODMAP diet’s effectiveness, customization, and flexibility make it a promising option for those seeking relief from IBS-related discomfort. Notably, research has predominantly focused on IBS-D patients, leaving gaps in our understanding, especially regarding microbiome implications and long-term effects, particularly in the second and third phases of the diet. Over the last two decades, research has progressed in tandem with revisions to the definition of IBS. Although information regarding long-term effectiveness and adherence is currently restricted, initial findings from observational studies hold promise.

It is imperative to undertake the FODMAP diet under the guidance of a healthcare professional to ensure a well-balanced and nutritionally adequate strategy. Further research is needed to deepen our understanding of the long-term implications and benefits of the FODMAP diet in managing IBS.
